# Bovine endometrial cells do not mount an inflammatory response to *Leptospira*

**DOI:** 10.1530/RAF-21-0012

**Published:** 2021-07-13

**Authors:** Paula C C Molinari, Jarlath E Nally, John J Bromfield

**Affiliations:** 1 Department of Animal Sciences, University of Florida, Gainesville, Florida, USA; 2 Infectious Bacterial Diseases Research Unit, National Animal Disease Center, Agricultural Research Service, United States Department of Agriculture, Ames, Iowa, USA

**Keywords:** leptospirosis, cattle, reproduction, inflammation, endometrium

## Abstract

Leptospirosis causes abortion, premature birth, and stillbirth in cattle, but the mechanisms remain unclear. Infected cattle shed *Leptospira* intermittently and present a range of clinical symptoms, making diagnosis difficult. The primary route of *Leptospira* transmission in any animal is the colonization of the renal tubule and excretion by urine; however, *Leptospira* can also colonize the female reproductive tract of cows and can be transmitted by semen. Vaccination against *Leptospira* in the US is routine in cattle, but immunity is not guaranteed. The cell wall of *Leptospira* contains toll-like receptor agonists including peptidoglycan and lipopolysaccharide. The capacity of *Leptospira* to initiate an innate inflammatory response from uterine endometrial cells is unknown but may be a cause of reproductive failure. Using cell culture, we tested the capacity of bovine endometrial epithelial cells or human monocytes to elicit an inflammatory response to *Leptospira borgpetersenii* serovar Hardjo strain TC273. Cells were exposed to either heat-killed *Leptospira, Leptospira* outer membrane, *Escherichia coli* lipopolysaccharide, Pam3CSK4 or medium alone for 2 to 24 h. Exposure of bovine endometrial epithelial cells or human monocytes to heat-killed *Leptospira* or *Leptospira* outer membrane did not induce the expression of *IL1A*, *IL1B*, *IL6,* or *CXCL8*, while exposure to *E. coli* lipopolysaccharide or Pam3CSK4 increased the expression of *IL1A*, *IL1B*, *IL6,* and *CXCL8* compared to control cells. This data suggest that *Leptospira* does not trigger a classical inflammatory response in endometrial cells. Understanding the interaction between *Leptospira* and the female reproductive tract is important in determining the mechanisms of Leptospirosis associated with reproductive failure.

**Lay summary:**

Cows infected with the *Leptospira* have abortion and stillbirth. It is not known how *Leptospira* causes pregnancy failure in the cow. We tested if *Leptospira* causes inflammation in cells of the uterus which triggers pregnancy failure. We collected cells from the uterus of healthy cows at the abattoir and placed them into culture with *Leptospira* and measured the expression of genes associated with inflammation. To our surprise, cells of the uterus did not respond to *Leptospira*; however, the same cells did respond to other disease-causing bacteria found in the uterus. This suggests that cells of the uterus can recognize bacteria and produce an inflammatory reaction but not in response to *Leptospira*. This finding suggests the immune system of the uterus cannot detect *Leptospira* which may go on to cause reproductive failure in cows. Understanding how *Leptospira* interact with cells of the uterus will help reduce pregnancy failure of cows with leptospirosis.

## Introduction

Leptospirosis is a worldwide bacterial zoonotic disease caused by pathogenic species of *Leptospira*. Leptospirosis affects over 1 million people annually, resulting in 58,900 deaths (Costa *et al.* 2015, Torgerson *et al.* 2015). Numerous mammalian species serve as hosts for *Leptospira* including rodents and cattle (Nally *et al.* 2016). *Leptospira* infection occurs after exposure to environments contaminated by spirochetes, and the subsequent colonization of the renal tubule that results in intermittent excretion of spirochetes in the urine and further contamination of the environment. Infected hosts may be asymptomatic or present a variety of clinical symptoms including fever, liver failure, respiratory distress, and reproductive failure (Ellis 2015).

In cattle, the greatest impact of leptospirosis is abortion, stillbirth, premature birth, reproductive failure, and milk drop syndrome (Ellis 2015, Loureiro & Lilenbaum 2020). Cattle are susceptible to infection with multiple *Leptospira* species and serovars including *L. borgpetersenii* serovar Hardjo, *L. interrogans* serovar Pomona, *L. kirschneri* serovar Grippotyphosa, and *L. noguchii* (Miller *et al.* 1991, Martins *et al.* 2015, Nally *et al.* 2018). The most prominent species of *Leptospira* associated with reproductive failure in cattle is *L. borgpetersenii* serovar Hardjo which decreases conception rate and increases calving to conception interval (Ellis *et al.* 1981, Miller *et al.* 1991, Guitian *et al.* 1999, Rajeev *et al.* 2014). Vaccination of cattle to serovars Canicola, Grippotyphosa, Hardjo, Icterohaemorrhagiae, and Pomona is common in the US and is an effective measure to prevent losses due to abortion and milk production. However, vaccination does not prevent infection and renal colonization, which results in vaccinated animals becoming carriers of *Leptospira* (Hanson et al. 1972, Srivastava 2006). In addition, host responses vary following vaccination suggesting a lack of complete and long-lasting protection, especially to *L. borgpetersenii* serovar Hardjo (Bolin et al. 1989, 1991, Rodrigues et al. 2011). A large proportion of the United States cattle herd is seropositive for pathogenic *Leptospira* (up to 49%), while seronegative cows can still excrete spirochete to transmit the infection to other animals (Miller *et al.* 1991, Talpada et al. 2003, Nally *et al.* 2018).

In cattle, leptospires have been isolated from the oviduct, uterus, aborted fetuses, and follicular fluid (Bielanski & Surujballi 1998, Bielanski *et al.* 1998, Monte *et al.* 2015, Loureiro & Lilenbaum 2020). Leptospires can migrate to the upper reproductive tract when administered intranasally or intracervically (Bielanski & Surujballi 1998, Bielanski *et al.* 1998). Interestingly, the detection of *Leptospira* in vaginal mucus by PCR is poorly correlated with detection in urine (Loureiro *et al.* 2017, Pinna *et al.* 2018). While leptospirosis causes reproductive failure in cattle, the mechanism by which this occurs is yet to be determined. It has been suggested that endometrial inflammation caused by *Leptospira* can change the developmental environment of the early conceptus, rendering the uterus hostile to pregnancy and resulting in reproductive failure (Loureiro & Lilenbaum 2020).

Postpartum uterine infection caused by gram-negative and gram-positive bacteria reduces reproductive success and causes localized inflammation of the endometrium in cattle. Epithelial and stromal cells of the endometrium respond to bacterial cell wall components, including lipopolysaccharide (LPS), via the toll-like receptor family and increase the expression of pro-inflammatory mediators including *IL1B*, *IL6,* and *CXCL8* (Cronin *et al.* 2012, Turner *et al.* 2014). We hypothesized that bovine endometrial cells elicit an innate immune response to *L. borgpetersenii* serovar Hardjo. In addition, we aimed to determine the rate of *Leptospira* infection of cows by sampling urine, blood, and the uterus of vaccinated cows. Understanding the endometrial response to *Leptospira* infection will increase our knowledge of how reproductive failure occurs in cows with leptospirosis.

## Materials and methods

### Antigen preparation and immunoblotting

*Leptospira borgpetersenii* serovar Hardjo strain TC273 was isolated from a bovine urine sample in Iowa, as previously described (Nally *et al.* 2018). The virulence of TC273 was evaluated by intraperitoneal injection into Syrian hamsters (*Mesocricetus auratus*) as described by Nally *et al.* (2018). Outer-membrane fractions of low-passage virulent *Leptospira borgpetersenii* serovar Hardjo strain TC273 were enriched using Triton-X114 as previously described (Nally *et al.* 2001). Outer membrane (OM)-enriched fractions were compared to heat-killed leptospires by 1-D gel electrophoresis as previously described (Monahan *et al.* 2008). Total proteins were visualized by staining with Sypro Ruby (Invitrogen), and lipopolysaccharide was visualized by staining with Pro-Q Emerald 300 (Invitrogen) as per the manufacturer’s guidelines. For immunoblotting, samples were transferred to Immobilon-P transfer membrane (Millipore) and blocked overnight at 4°C with StartingBlock (TBS) blocking buffer (Thermo Fisher Scientific). Membranes were individually incubated with indicated antisera (anti-LipL21, anti-LipL32, and anti-LipL41 at 1:2500, 1:4000, and 1:2500, respectively or anti-Hardjo at 1:2500) in PBST for 1 h at room temperature, followed by incubation with horseradish-peroxidase anti-rabbit immunoglobulin G conjugate (Sigma–Aldrich). Bound conjugates were detected using Clarity Western ECL substrate (Bio-Rad Laboratories), and images were acquired using a Bio-Rad ChemiDoc MP imaging system.

### Bovine endometrial epithelial cell culture

Bovine endometrial epithelial (BEND) cells were obtained from the American Type Culture Collection (ATCC, Manassas, VA; CRL-2398). Cells were cultured in complete culture medium (40% Ham F-12, 40% MEM, 10% fetal bovine serum, 10% horse serum, 100 IU/mL penicillin, 100 μg/mL streptomycin, 1.5 g/L of sodium bicarbonate, 0.034 mg/mL D-valine; Thermo Fisher Scientific) in 75 cm^2^ flasks (Greiner Bio-One, Monroe, NC) at 38.5°C in a humidified atmosphere containing 95% air and 5% CO_2_ until subconfluent. Cells were seeded on 24-well plates (TPP, Trasadingen, Switzerland) at a final density of 10^5^ cells/well in 500 μL and equilibrated for 24 h before the addition of treatments. Each experiment was performed seven times, with each replicate utilizing BEND cells between passages 3 and 13.

### Human THP-1 cell culture

The human monocyte cell line, THP-1 (ATCC; TIB202) was used as a positive control for all treatments. THP-1 cells are a specialized immune cell line that elicits a strong innate immune response to pathogen-associated molecules, including *Escherichia coli-*derived LPS. Cells were cultured in 75 cm^2^ flasks (Greiner Bio-One) in a complete culture medium containing RPMI 1640, 10% fetal calf serum, and 0.05 mM 2-mercaptoethanol (Thermo Fisher Scientific) at 37°C in a humidified atmosphere containing 95% air and 5% CO_2_ until subconfluent. Cells were plated in 24-well plates (TPP) at a density of 10^5^ cells/mL in 500 uL and incubated for 48 h in the presence of 50 ng/mL phorbol myristate acetate to promote the differentiation of monocytes to macrophage-like cells. Treatments were applied to THP-1 cells after differentiation. Each experiment was performed four times, with each replicate utilizing THP-1 cells between passages 12 and 14.

### Treatment of cultured cells

Adherent THP-1 and BEND cells were exposed for 24 h to either ultrapure *E. coli* O111:B4 LPS (Invivogen), Pam3CSK4 (Invivogen), heat-killed *Leptospira borgpetersenii* serovar Hardjo (HK-lepto), *Leptospira borgpetersenii* serovar Hardjo outer membrane preparation (OM-lepto) or medium alone as a control. Doses of each treatment started at 10,000 ng/mL and decreased ten-fold to 1 ng/mL (maximal HK-lepto and OM-lepto treatments were 1000 ng/mL). To determine acute cellular responses, THP-1 and BEND cells were exposed to 100 ng/mL of LPS, Pam3CSK4, HK-lepto, OM-lepto or control medium for 2 or 12 h. Cell-free supernatants were collected, and total cellular RNA was stabilized in 350 μL of RLT buffer (Qiagen) and stored at −80°C.

### Evaluation of endometrial cell viability

Viability of BEND cells was assessed by the cellular reduction of MTT (Thermo Fisher Scientific) as previously described (Rizo *et al.* 2019). Briefly, BEND cells were cultured in 96-well culture plates (TPP) at a density of 10^5^ cells/mL in 200 μL and equilibrated for 24 h at 38.5°C in a humidified atmosphere containing 95% air and 5% CO_2_. Duplicate wells were exposed to 100 ng/mL of LPS, Pam3CSK4, HK-lepto, OM-lepto, or control medium for 2 or 24 h. Following treatment, 10 uL of MTT (5 mg/mL) was added to each well and incubated at 38.5°C for 4 h. Cells were washed with warm DPBS and lysed in 100 μL of dimethyl sulfoxide. Optical density of each well was measured using a microplate reader at 540 nm (BioTek). The blank corrected value for each well was determined, and an average optical density for each replicate was calculated using the average of duplicate wells. Data were normalized and expressed as fold change from cells treated with control culture medium only.

### RNA extraction and real-time RT-PCR

Total RNA was extracted using the RNeasy Mini kit (Qiagen) according to the manufacturer’s instructions. Samples were quantified and checked for RNA quality by spectrophotometry (Nanodrop ND1000, Thermo Fisher Scientific) and then reverse-transcribed using the Verso cDNA kit according to the manufacturer’s instructions (Thermo Fisher Scientific). Primers were designed using the NCBI primer-design tool and verified by BLAST with the exception of human IL1A that was obtained from Sharkey *et al*. (2012) (Table 1). All primers were validated for amplification efficiency prior to sample analysis and conformed to MIQE guidelines (Pearson correlation coefficient r^2^ > 0.98 and efficiency between 90 and 110%) (Bustin *et al.* 2009). Real-time PCR was performed in 20 µL reactions, containing 18 µL of SYBR Green Master Mix (Bio-Rad Laboratories) with 500 nM of each reverse and forward primer and 2 µL of template cDNA. The cycling was performed on a CFX Connect Real-Time PCR System (Bio-Rad Laboratories) consisting of an initial denaturation/enzyme activation step for 30 s at 95°C followed by 39 PCR cycles using 5 s denaturation at 95°C, 30 s annealing at 60°C, and 10 s of extension at 65°C. Each reaction was performed in duplicate. A no-template control with no cDNA was included for each primer set to demonstrate the absence of non-specific amplification. Relative expression for genes of interest was calculated using the 2^−ΔCt^ method relative to *GAPDH* for BEND cells and *ACTB* for THP-1 cells (Livak & Schmittgen 2001). Expression of *GAPDH* and *ACTB* was stable (*P* > 0.05) across all treatments (Supplementary Fig. 1, see section on supplementary materials given at the end of this article).

**Table 1:** PCR primers sequences used for real-time RT-PCR.

Gene symbol	Sequence (5’–3’)		Accession number
	Forward	Reverse	
Human			
*ACTB*	ACAGAGCCTCGCCTTTGCCG	TTGCACATGCCGGAGCCGTT	NM_001101.3
*CXCL8*	CAGAGACAGCAGAGCACACA	GGCAAAACTGCACCTTCACA	NM_000584.3
*IL1A*	CCAACGGGAAGGTTCTGAAG	GGCGTCATTCAGGATGAATTC	NM_000575
*IL1B*	AACCTCTTCGAGGCACAAGG	GTCCTGGAAGGAGCACTTCAT	NM_000576.2
*IL6*	CAGTTCCTGCAGAAAAAGGCAA	GCTGCGCAGAATGAGATGAG	NM_000600.3
Bovine			
*CXCL8*	GCAGGTATTTGTGAAGAGAGCTG	CACAGAACATGAGGCACTGAA	NM_173925.2
*GAPDH*	AGGTCGGAGTGAACGGATTC	ATGGCGACGATGTCCACTTT	NM_001034034.2
*IL1A*	AGAGGATTCTCAGCTTCCTGTG	ATTTTTCTTGCTTTGTGGCAAT	NM_174092
*IL1B*	CTTCATTGCCCAGGTTTCTG	CAGGTGTTGGATGCAGCTCT	NM_174093.1
*IL6*	ATGACTTCTGCTTTCCCTACCC	GCTGCTTTCACACTCATCATTC	NM_173923.2

### Tissue and fluid collection to determine the prevalence of *Leptospira* spp in blood, urine, and the uterus

The University of Florida Institutional Animal Care and Use Committee approved all animal procedures that were conducted at the University of Florida Dairy Research Unit. Two distinct cohorts of vaccinated cows were used for the collection of blood, urine, and uterine samples. All sampled cows were vaccinated (Bovi-Shield Gold FP 5 VL5 HB or CattleMaster Gold FP 5 L5, Zoetis, Parsippany, NJ) against bovine viral diarrhea, infectious bovine rhinotracheitis, parainfluenza, bovine respiratory syncytial virus, and multiple serovars of *Leptospira* including serovar Hardjo, Pomona, Grippotyphosa, Canicola, and Icterohemorrhagiae.

Cohort 1 consisted of 33 lactating Holstein cows in lactation 1 to 5, born and raised at the University of Florida Dairy Research Unit. Cohort 2 consisted of 23 2-year old primiparous non-lactating Holstein cows born and raised in South Georgia and housed at the University of Florida Dairy Research Unit after calving.

Urine samples were collected from all 56 cows in cohorts 1 and 2 to determine the presence of *Leptospira spp* in the urinary tract by fluorescent antibody test (FAT) and culture (see below for details). Approximately 30 mL of midstream urine was collected from each cow into a sterile 50 mL conical tube after stimulation of the perineal area. Urine samples were immediately placed on ice and processed for live culture within 1 h of collection by adding two to three drops of fresh urine to individual culture tubes containing culture medium as previously described by Nally *et al.* (2018). The remaining urine was utilized for FAT analysis. Postmortem urine was collected from one cow in cohort 2, 63 days after the first urine collection. Within 30 min of slaughter, a sterile needle and syringe were used to aspirate urine from the bladder which was transferred to a sterile vial. The vial was maintained on ice for detection of *Leptospira spp* by FAT and live culture.

Whole blood was collected from the coccygeal vessel into evacuated tubes (Vacutainer, Becton Dickson, Franklin Lakes, NJ) without anticoagulant from six cows in cohort 2 prior to slaughter. Blood was collected 63 days after initial urine sampling. Whole blood was centrifuged for 10 min at 2400 ***g*** at room temperature, and subsequent serum was frozen at −20°C. Serum samples were used to evaluate the exposure of cows to *Leptospira* antigen using the microscopic agglutination test (MAT; see below for details).

Uterine samples from cows in cohorts 1 and 2 were collected using the cytobrush technique immediately after initial urine collection as previously described (Rizo *et al.* 2019). Briefly, external genitalia were cleaned using 70% ethanol, and the cytobrush tool (Medscan Medical, Cooper Surgical, Trumball, CT) contained inside a metal sheath and covered by a sanitary chemise (WTA, Cravinhos, Brazil) was introduced into the vagina. Using rectal palpation, the tool was passed through the cervix and the sanitary chemise was retracted over the metal sheath. The cytobrush was then extended and placed in direct contact with the endometrium. The cytobrush was then rotated three times to sample endometrial cells and uterine fluid. The cytobrush was then retracted into the metal sheath and removed from the reproductive tract. Endometrial smears were prepared by gently rolling the cytobrush over a clean, glass slide (Thermo Fisher Scientific) and air-dried. Uterine samples were collected from a total of 27 cows and used for detection of *Leptospira* by FAT.

### Fluorescence antibody test to determine the presence of *Leptospira* spp

The FAT was performed as previously described (Nally *et al.* 2018). Briefly, urine samples were centrifuged at 10,000 ***g*** for 30 min at 4°C. The resultant cell pellet was resuspended in 2 mL H_2_O and 1 mL was transferred to a clean 1.5 mL tube and washed by centrifugation at 12,000 ***g*** for 10 min at 4°C. Approximately 100 μL of material was retained after centrifugation and was diluted in 500 μL of H_2_O and centrifuged at 12,000 ***g*** for 10 min at 4°C. The supernatant was removed until approximately 50 μL remained which was then resuspended. A 15 μL aliquot of the resultant suspension was deposited on a 7 mm well FAT glass slide in duplicate. Slides were air-dried and fixed in acetone for 10 min and placed in a humidified chamber. Each well was incubated at 37°C for 1 h with 20 μL of high-titer rabbit anti-*Leptospira* sera (National Veterinary Services Laboratories, APHIS, USDA, Ames, IA) conjugated to fluorescein isothiocyanate. Slides were washed in PBS with gentle rocking for 10 min, dried and counterstained with Flazo Orange (National Veterinary Services Laboratory), followed by a final wash in PBS before coverslips were mounted using Vectashield (Vector Laboratories, Burlingame, CA). Microscopic examination was performed using a Nikon Eclipse E800 microscope and B2-A filter (excitation, 450–490 nm; emission, 520 nm) at 200× magnification. Positive FAT samples were categorized by the fluorescein isothiocyanate fluorescence that conformed to *Leptospira* morphology.

### Microscopic agglutination test

Serum of six cows in cohort 2 was evaluated for the presence of antibodies to six live *Leptospira* serovars; *L. interrogans* serogroup Australis serovar Bratislava strain Jez Bratislava, *L. interrogans* serogroup Canicola serovar Portlandvere strain 12-001, *L. kirschneri* serogroup Grippotyphosa serovar Grippotyphosa strain GR-01-082, *L. interrogans* serogroup Sejroe serovar Hardjo strain Hardjoprajitno, *L. interrogans* serogroup Icterohemorrhagiae serovar Copenhageni strain IC-02-001, and *L. interrogans* serogroup Pomona serovar Pomona strain Pomona. Briefly, sample serum was added to live *Leptospira* cultures at two-fold increasing dilutions beginning at 1:25, up to 1:800.

### Statistical analysis

Data were analyzed using SPSS software V24.0 (IBM Analytics). Data were assessed for normality with Shapiro–Wilk test and log transformed when appropriate (described in figure legends). Data were analyzed using the generalized linear mixed model with dose, treatment, and time as fixed factors. Pairwise comparisons were performed between each dose and vehicle controls. Statistical significance was declared when *P* ≤ 0.05.

## Results

### Characterization of *Leptospira* preparations used for cell treatments

**Figure 1:**
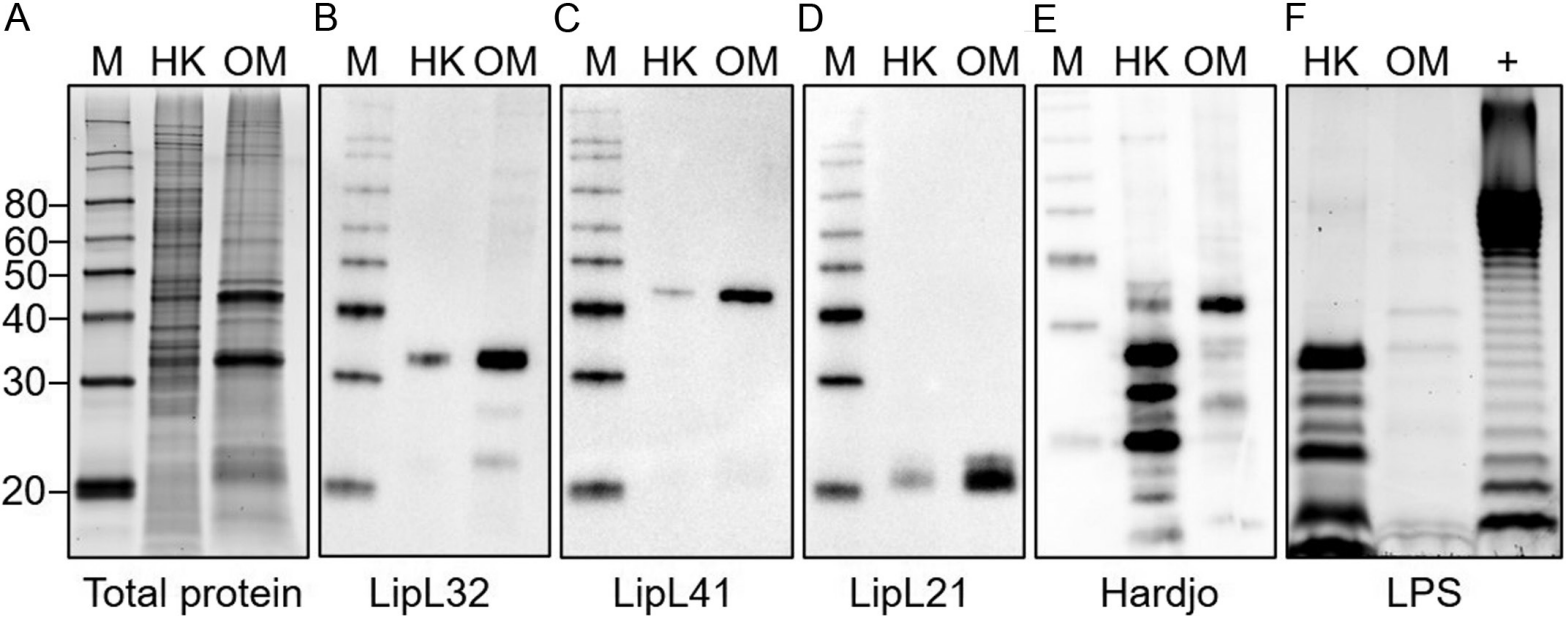
Characterization of heat-killed and outer membrane extract of *Leptospira borgpetersenii*. Total protein stain (A) of 4 µg of heat-killed (HK) *Leptospira borgpetersenii* serovar Hardjo strain TC273 or outer membrane (OM) proteins of *L. borgpetersenii* serovar Hardjo strain TC273. Immunoblots of 0.5 µg HK or OM probed with antiserum specific for protein LipL32 (B), the outer membrane lipoprotein LipL41 (C), and the outer membrane lipoprotein LipL21 (D). HK of 4 µg or OM stained for the presence of LPS (E), including a positive control (+) of 5 µg of *E. coli* serotype 055: B55 LPS. Molecular mass marker is labeled ‘M’. A total of 0.5 µg HK or OM was probed using anti-Hardjo antiserum (F).

Heat-killed *Leptospira borgpetersenii* serovar Hardjo (HK-lepto) and *L. borgpetersenii* serovar Hardjo outer membrane preparation (OM-lepto) used to treat BEND cell and THP-1 cells were evaluated for the presence of *Leptospira* specific proteins and LPS after completion of cell stimulation experiments to confirm bioactivity (Fig. 1). Both preparations of HK-lepto and OM-lepto showed the presence of LipL32 (32kDa; Fig. 1B), LipL41 (41 kDa; Fig. 1C), LipL21 (21kDa; Fig. 1D), and reactivity to anti-Hardjo antiserum (Fig. 1E). Both preparations of HK-lepto and OM-lepto showed the presence of LPS using the Pro-Q Emerald 300 LPS stain (Fig. 1F), in an expected different conformation than *E. coli* 055: B55 LPS.

### Effect of bacterial components in endometrial cell viability

**Figure 2:**
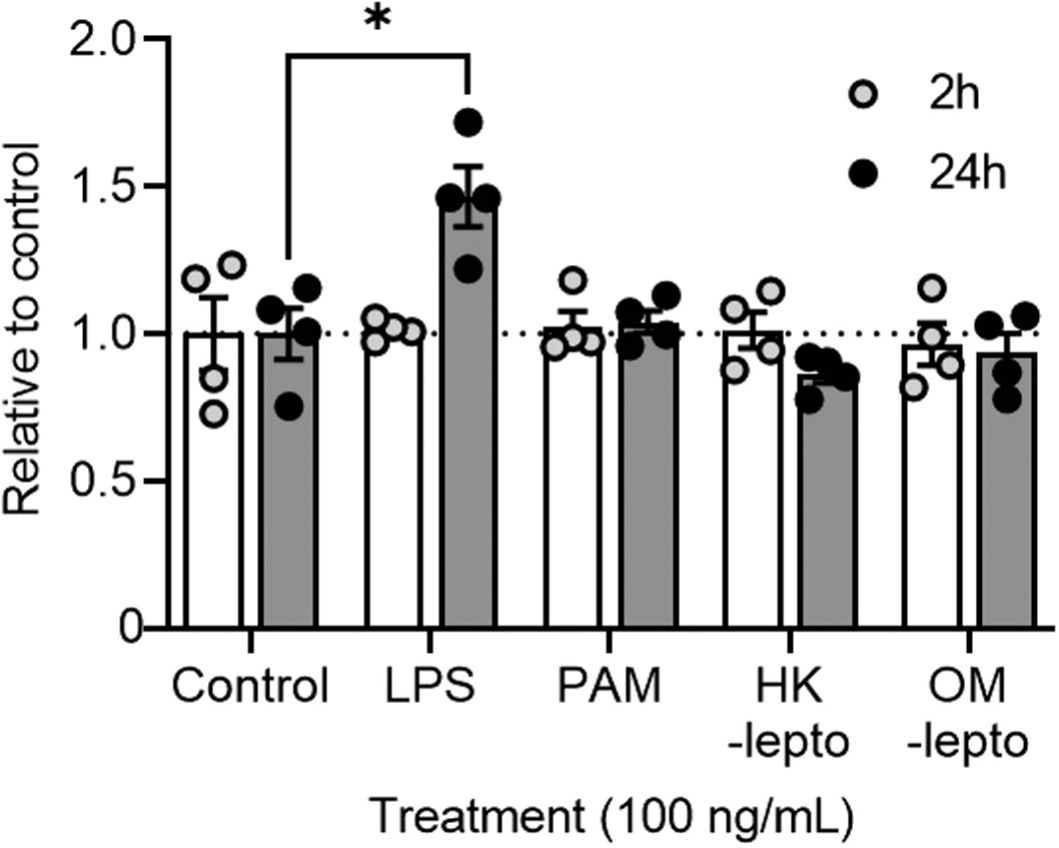
Cell viability after exposure to bacterial components. Cell viability was assessed in BEND cells after 2 or 24 h exposure to 100 ng/mL of *E. coli* LPS, Pam3CSK4, heat-killed *Leptospira* (HK-lepto), or *Leptospira* outer membrane preparation (OM-lepto). Cell viability is expressed relative to medium alone only controls. Each time point consisted of four independent replicates. Bars represent the mean ± s.e.m., and dots represent individual replicates. * *P* ≤ 0.05.

Exposure of BEND cells to LPS, Pam3CSK4, HK-lepto or OM-lepto for 2 h had no effect on cell viability compared to controls (Fig. 2). While exposure of BEND cells to Pam3CSK4, HK-lepto, or OM-lepto for 24 h did not affect cell viability, exposure to LPS for 24 h increased cell viability compared to medium only controls (*P <* 0.05).

### Effect of bacterial components on the expression of inflammatory mediators in cultured cells

**Figure 3:**
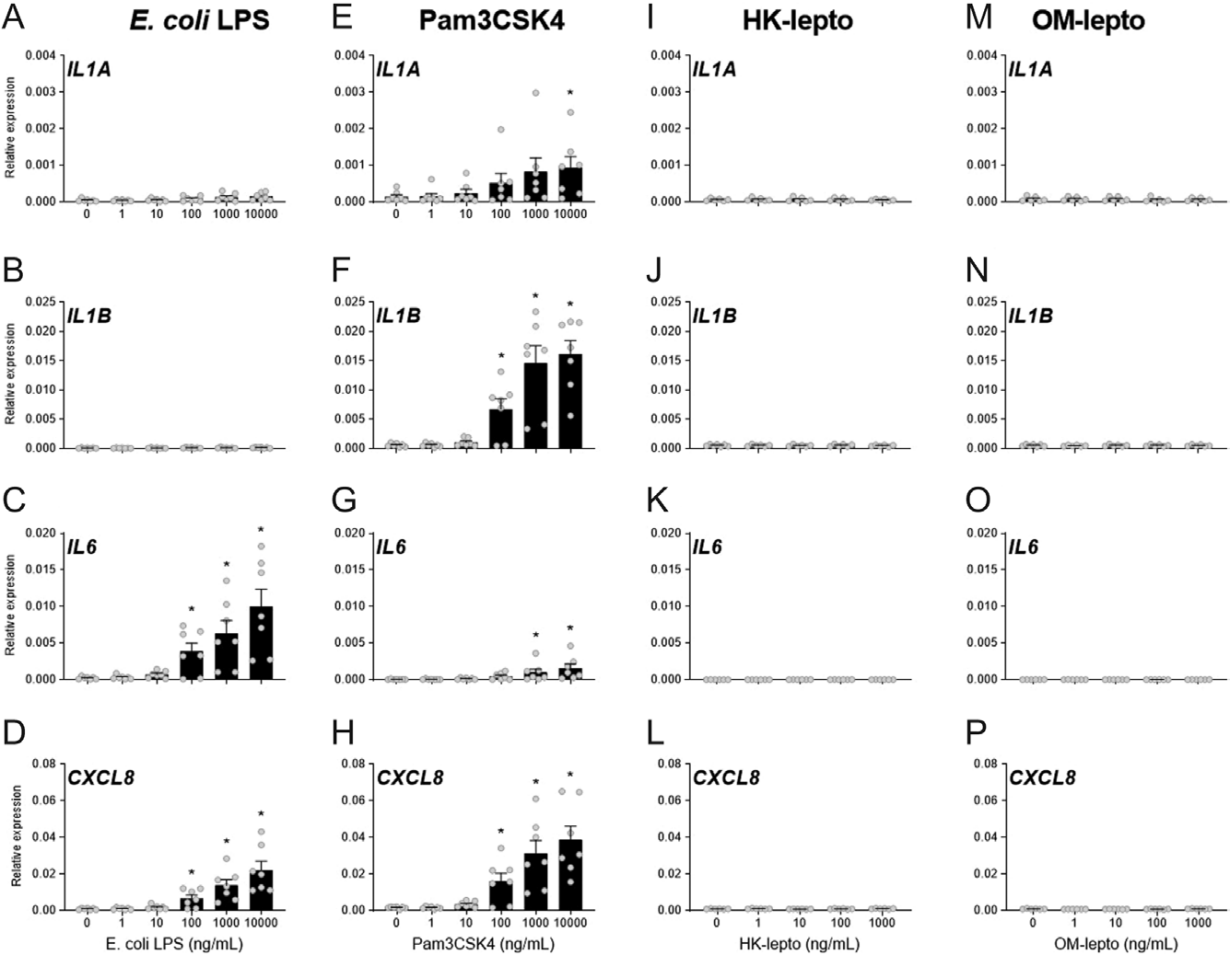
Response of BEND cells to bacterial components. BEND cells were exposed to ultrapure *E. coli* LPS (A - D), Pam3CSK4 (E - H), heat-killed *Leptospira* (HK-lepto; I - L), *Leptospira* outer membrane preparation (OM-lepto; M - P) or control medium for 24 h. Expression of the inflammatory mediators *IL1A*, *IL1B*, *IL6,* and *CXCL8* is expressed relative to *GAPDH*. Each treatment was repeated in seven independent replicates. Bars represent the mean ± s.e.m., and dots represent individual replicates. * *P* ≤ 0.05 compared to medium alone controls following Tukey’s test.

Exposure of BEND cells to 100, 1000, or 10,000 ng/mL of LPS for 24 h increased the expression of *IL6* and *CXCL8* compared to medium alone controls (Fig. 3C and D; *P* < 0.05). Exposure to 100, 1000, or 10,000 ng/mL of Pam3CSK for 24 h increased BEND cell expression of *IL1B* and *CXCL8* compared to medium alone controls, while 1000 or 10,000 ng/mL of Pam3CSK4 increased the expression of *IL6* compared to medium alone controls and exposure to 10,000 ng/mL of Pam3CSK4 increased the expression of *IL1A* compared to medium alone controls (Fig. 3E, F, G and H; *P* < 0.05). Exposure of BEND cells to either HK-lepto or OM-lepto did not alter the expression of *IL1A*, *IL1B*, *IL6,* or *CXCL8* compared to medium alone controls (Fig. 3I, J, K, L, M, N, O and P).

**Figure 4:**
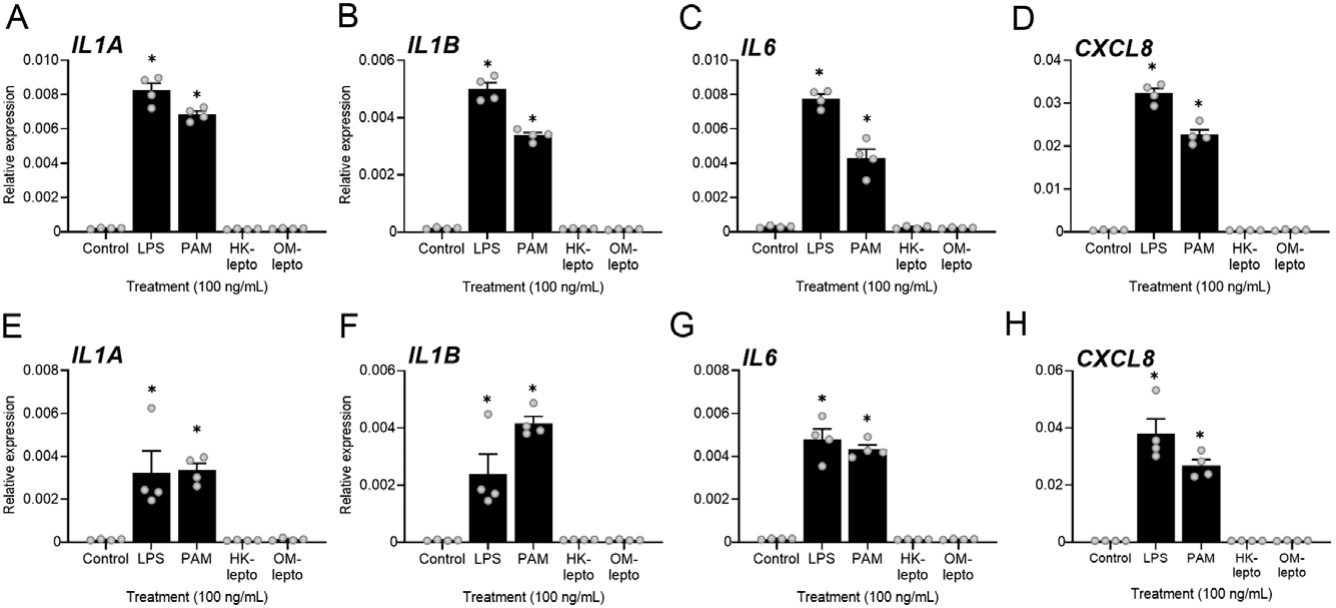
Acute response of BEND cells to bacterial components. BEND cells were exposed to ultrapure *E. coli* LPS, Pam3CSK4, heat-killed *Leptospira* (HK-lepto), *Leptospira* outer membrane preparation (OM-lepto), or control medium for 2 (A - D) or 12 (E - H) h. Expression of the inflammatory mediators *IL1A*, *IL1B*, *IL6,* and *CXCL8* is expressed relative to *GAPDH*. Each treatment was repeated in four independent replicates. Bars represent the mean ± s.e.m., and dots represent individual replicates. * *P* ≤ 0.05 compared to medium alone controls following Tukey’s test.

Acute exposure of BEND cells to 100 ng/mL of LPS or Pam3CSK for 2 or 12 h increased the expression of *IL1A*, *IL1B*, *IL6,* and *CXCL8* compared to medium alone controls (Fig. 4A,B, C, D, E, F, G and H; *P* < 0.05). However, exposure of BEND cells to either HK-lepto or OM-lepto for 2 or 12 h did not alter the expression of IL1A, *IL1B*, *IL6,* or *CXCL8* compared to medium alone controls (Fig. 4A,B, C, D, E, F, G and H).

**Figure 5:**
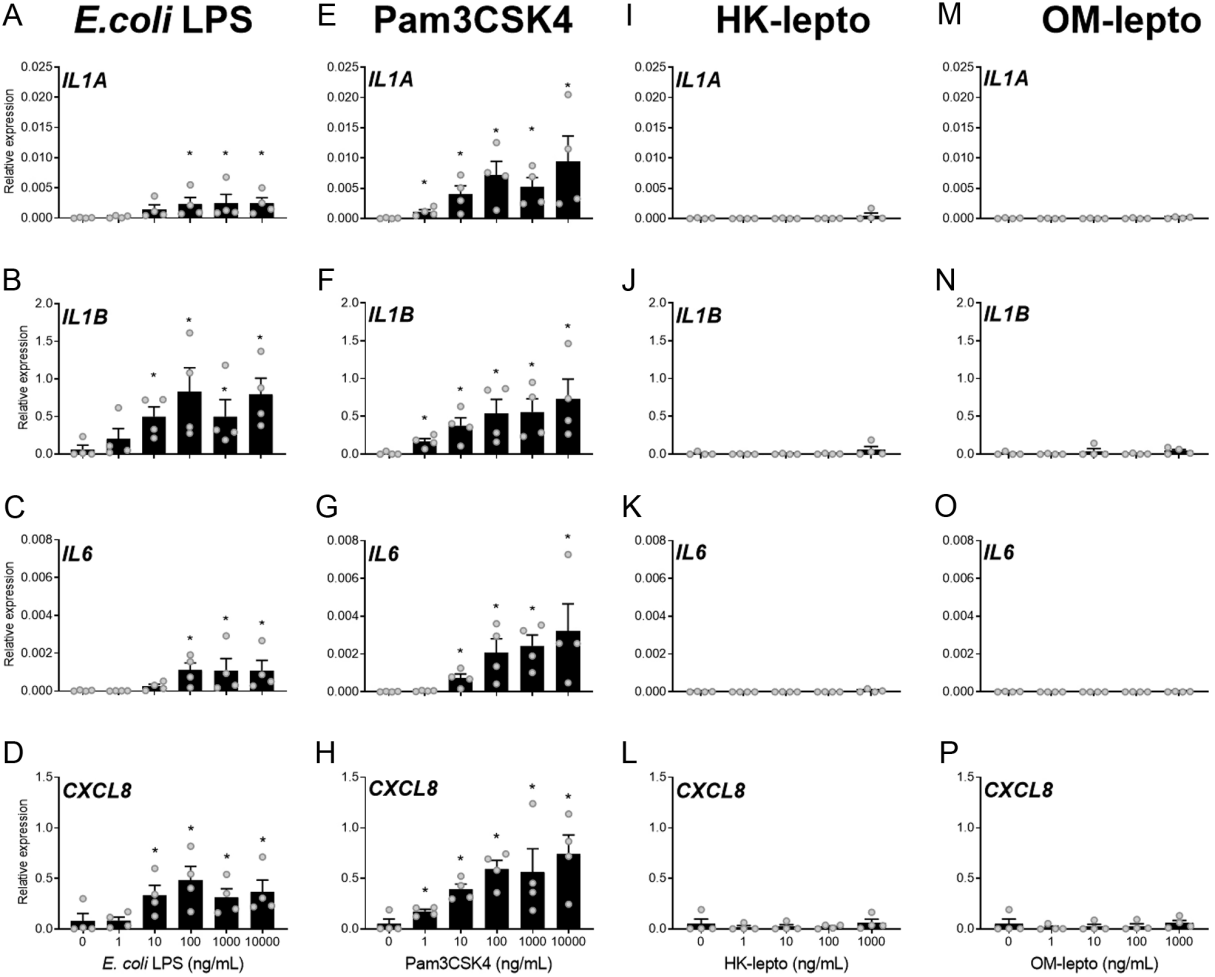
Response of THP-1 cells to bacterial components. Gene expression of activated THP-1 cells exposed for 24 h to ultrapure *E. coli* LPS (A - D), Pam3CSK4 ( E - H), heat-killed *Leptospira* (HK-lepto; I - L) or *Leptospira* outer membrane preparation (OM-lepto; M - P). Expression of the inflammatory mediators *IL1A*, *IL1B*, *IL6,* and *CXCL8* is expressed relative to *ACTB*. Each treatment was repeated in four independent replicates. Bars represent the mean ± s.e.m., and dots represent individual replicates. * *P* ≤ 0.05 compared to medium alone controls following Tukey’s test.

Exposure of THP-1 cells to 10, 100, 1000, or 10,000 ng/mL of LPS for 24 h increased the expression of *IL1B* and *CXCL8* compared to medium alone controls, while exposure to 100, 1000, and 10,000 ng/mL of LPS for 24 h increased the THP-1 expression of *IL1A* and *IL6* (Fig. 5A,B, C and D; *P* < 0.05). Exposure of THP-1 cells to 1, 10, 100, 1000, or 10,000 ng/mL of Pam3CSK for 24 h increased the expression of *IL1A*, *IL1B,* and *CXCL8* compared to medium alone controls, while exposure to 10, 100, 1000, or 10,000 ng/mL of Pam3CSK for 24 h increased the THP-1 cell expression of *IL6* compared to medium alone controls (Fig. 5E, F, G and H; *P* < 0.05). Exposure of THP-1 cells to either HK-lepto or OM-lepto for 24 h did not alter the expression of *IL1A*, *IL1B*, *IL6,* or *CXCL8* compared to medium alone controls (Fig. 5I, J, K, L, M, N, O and P).

### Detection of *Leptospira* in urine and uterine samples

**Figure 6:**
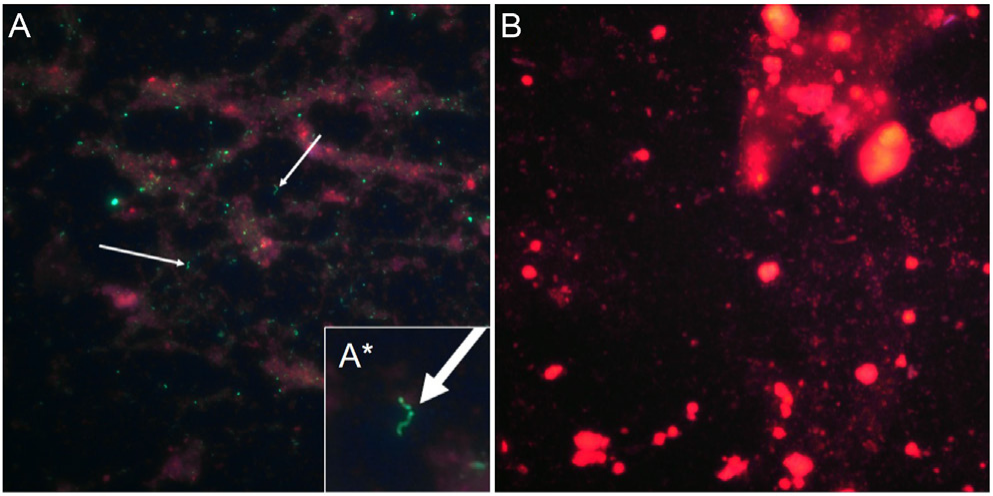
Detection of *Leptospira* in urine using fluorescent antibody test. Urine and uterine samples were subjected to a fluorescent antibody test (FAT) to detect the presence of the *Leptospira*-specific protein LipL32. Urine samples (A) showing the presence of LipL32 immunoreactivity and classical *Leptospira* morphology. Insert (A*) is a higher magnification showing the *Leptospira* morphology. A *Leptospira* negative urine sample is shown in (B). Arrows highlight LipL32 positive structures with *Leptospira* morphology. Images are collected at 200× magnification.

Urine collected by micturition from 56 cows was analyzed for the presence of *Leptospira spp* using FAT. Urine of one cow (1.8%) in cohort 2 was FAT-positive that showed a *Leptospira* morphology (Fig. 6). Postmortem urine from the bladder of this single positive cow was collected 63 days after the first urine collection and was FAT-negative. All urine samples were culture negative.

All uterine samples collected from 27 cows by cytobrush (including the urine FAT-positive cow) were FAT negative.

### Microscopic agglutination test of serum

Serum of six cows in cohort 2 was used to determine the presence of *Leptospira* antibodies 63 days after the first urine FAT analysis (Table 2). Blood from the single FAT-positive cow (cow #1) was included in the evaluation. Of the six cows tested, three had a positive MAT of 1:25 or greater against one serovar, including Canicola and Grippotyphosa. The single urine FAT-positive cow was MAT positive for Canicola, while two FAT-negative cows were MAT positive for Grippotyphosa.

**Table 2:** MAT results from sera collected from group 2.

Cow	Serovar					
	*Bratislava*	*Canicola*	*Grippotyphosa*	*Hardjo*	*Icterohaenorrhagiae*	*Pomona*
1	-	1:25	-	-	-	-
2	-	-	1:25	-	-	-
3	-	-	-	-	-	-
4	-	-	-	-	-	-
5	-	-	-	-	-	-
6	-	-	1:25	-	-	-

1:25, agglutination of sample at 1:25 serum dilution; -, no agglutination.

## Discussion

Leptospirosis in cattle is common and causes several clinical complications including reproductive failure. While the mechanisms of leptospirosis-induced reproductive failure are unknown, spirochetes are located within the female reproductive tract and it has been proposed that uterine inflammation caused by *Leptospira* could result in reproductive failure (Loureiro & Lilenbaum 2020). Both gram-negative and gram-positive bacteria (and isolated components) induce TLR signaling in bovine endometrial cells that results in an innate immune response to pathogens with increased expression of inflammatory mediators including IL1A, *IL1B*, *IL6,* and *CXCL8* (Cronin *et al.* 2012, Turner *et al.* 2014). Bovine endometrial epithelial and stromal cells express all ten TLRs and when activated by pathogens increase the expression of cytokines and chemokines to induce a cellular inflammatory response and clear the pathogenic agent (Davies *et al.* 2008, Cronin *et al.* 2012, Turner *et al.* 2014). Here, bovine endometrial epithelial cells and human monocytes increased the expression of *IL1A*, *IL1B*, *IL6,* and *IL8* when exposed to ultrapure gram-negative LPS (TLR4 agonist) or the synthetic lipopeptide Pam3CSK4 (TLR2/1 agonist). However, when either cell type was exposed to heat-killed *Leptospira* or purified outer membrane preparations of *Leptospira* for 2, 12, or 24 h no change in *IL1A*, *IL1B*, *IL6,* and *CXCL8* expression was observed. These data suggest that while bovine endometrial cells have the functional capacity to induce an inflammatory response to bacterial components, *Leptospira* either evade detection by endometrial epithelial cells or elicit a non-classical immune response that was not measured here.

Previous experimental data in female dogs and mares demonstrate that *Leptospira* infection induces endometrial inflammation and is associated with reproductive failure (Pinna *et al.* 2013, Wang *et al.* 2014). In parallel, exposure of bovine neutrophils to *Leptospira* induced minimal expression of inflammatory cytokines and slight neutrophil extracellular trap formation (Wilson-Welder *et al.* 2016). In addition, experimental inoculation of the uterus with *Leptospira* increased pregnancy failure in cattle and increased *Leptospira* titers in blood and vaginal mucus (Dhaliwal *et al.* 1996). Collectively, these studies suggest that peripheral cells or endometrial cells elicit an inflammatory response to *Leptospira*; however, other studies conclude that intrauterine inoculation of *Leptospira* has no effect on fertility or clinical inflammation, suggesting secondary mechanisms which may be responsible for reproductive failure in cattle (Vahdat *et al.* 1983). Indeed, *Leptospira* pathogens have been isolated from aborted fetuses and embryos of infected dams and as such may directly impact the reproductive success of the host by directly targeting the conceptus as opposed to the female reproductive tract (Ellis *et al.* 1981, Bielanski *et al.* 1998).

Most recently, molecular mechanisms have been described by which *Leptospira* can evade detection of TLR4, TLR5, NOD1, and NOD2 of the innate immune system (Werts *et al.* 2001, Ratet *et al.* 2017, Holzapfel *et al.* 2020). Our own characterization of LPS shown here (Fig. 1), clearly indicates that *E.coli* and *Leptospira* LPS are structurally different and result in different molecular weights. Indeed, previous work demonstrates that *E. coli* LPS induces a strong immune response in rabbits compared to LPS purified from *Leptospira* (de Souza & Koury 1992). The *Leptospira* outer membrane preparation used here contains LPS, LipL32, LipL41, LipL21, glycolipids, lipoproteins, and various other pathogen-associated molecular patterns that could be involved in virulence and induce a host immune response. In humans, *L. interrogans* can evade recognition by TLR4 and activates macrophages using TLR2 (Werts *et al.* 2001). The process of *Leptospira* immune system evasion may be specific to the growth stage of the pathogen and the species of the host (Holzapfel *et al.* 2020). When cattle are infected with *Leptospira,* the absence of clinical symptoms is common despite the continued excretion of *Leptospira* in urine and the presence of the pathogen in the reproductive tract. It is suggested that persistent infection is due to specific host-pathogen mechanisms that dampen the host immune response to resident *Leptospira*. However, when excreted in urine *Leptospira* modify their protein and antigen expression and modulate post-translational protein modifications which may aid in pathogen evasion of the host immune system (Nally *et al.* 2005, 2011). Understanding the molecular mechanisms by which *Leptospira* evades recognition by the host immune system require careful investigation in target species using specific cell types, and likely* in vivo* experimentation.

Studies estimate that up to 49% of cattle in the US are seropositive for pathogenic *Leptospira,* and vaccination to serovar Hardjo can induce an inconsistent response that results in short-term immunization (Miller *et al.* 1991). Our results screening urine and uterine samples of immunized animals by FAT and culture did not identify any *Leptospira-*positive cattle at the University of Florida herd. In general, all cattle at the University of Florida dairy research unit are born and raised on-site, providing for a closed system. However, we also screened cattle that were imported from Georgia, with one cow providing a positive urine FAT that was later negative when urine was collected from the bladder at the time of slaughter. This cow demonstrated intermittent shedding of *Leptospira* in urine that has been observed previously (Cordonin *et al.* 2020) and may suggest the presence of other *Leptospira-*positive cows that were not detected during the time of sampling. In addition, no uterine samples tested by FAT or culture returned a positive result; however, there is a poor correlation between the detection of *Leptospira* in urine and the uterus (Loureiro *et al.* 2017). In parallel, the same cows were tested for *Leptospira* infection by MAT at least 200 days after immunization to serovars Canicola, Gryppotyphosa, Hardjo, Icterohemorrhagiae, and Pomona. Considering the immunization status of the cows and inclusion of the single FAT-positive cow, MAT titers were surprisingly low or negative for tested cows. It is not clear from the very limited data presented here or from the available literature as to what MAT titers should be expected following *Leptospira* vaccination or *Leptospira* infection. It is important to note that the MAT assay is based on immunoglobulin (Ig) M that tends to increase rapidly after vaccination but rapidly declines and may, therefore, provide an inaccurate measure of immune status (Negi et al. 1971). The cows surveyed in this study were vaccinated at least 200 days prior to sample collection, which may explain the low titers observed in the MAT. Bacterin vaccines, like the ones used in the cows surveyed here, can produce a negative MAT response while still protecting the animal from *Leptospira* infection, which can be confirmed by assays that measure IgG, such as hamster immunization assays. Our data suggest that *Leptospira* serovar Hardjo does not induce a classical inflammatory response in bovine endometrial epithelial cells or human monocytes based on the specific inflammatory markers used here (*IL1A*, *IL1B*, *IL6,* and *CXCL8*) under the specific conditions tested. *Leptospira* may possess specific molecular mechanisms to evade detection by the innate immune system or perhaps induce a non-classical response with induction of other cytokines and mediators not measured here. It is also important to highlight that our model utilized a cell line and not isolated primary cells, thus extrapolation to an active *Leptospira* infection in the cow can only be considered with caution.

In cattle, the endometrial epithelial layer is commonly lost at the time of parturition which exposes the underlying stroma to bacterial pathogens that commonly cause postpartum uterine disease. Our culture model only evaluated the effects of *Leptospira* on epithelial cells and did not account for the possible interaction of *Leptospira* components on the underlying stroma. Moreover, the two-dimensional culture system employed here does not replicate epithelial cell polarization observed* in vivo*, and bovine endometrial epithelial cells do exhibit a vectorial release of specific inflammatory mediators when challenged by pathogens (Healy *et al*. 2015). Indeed, the current study fails to evaluate the effects of live *Leptospira* on endometrial cells, which would likely kill endometrial cells and induce subsequent inflammation via release of damage-associated molecular patterns. Nonetheless, the present study supports previous findings on how *Leptospira* might be able to evade host response and shows that might be true also for the bovine reproductive tract. Further investigation is required to determine if this evasion from the immune system plays a role in Leptospirosis-induced reproductive failure in cattle.

## Supplementary Material

Supplemental Figure 1. Expression of housekeeping genes in BEND and THP-1 cells. Expression of BEND cells GAPDH according to treatment (A-D) and expression of THP-1 cells ACTB according to treatment (E-H). Expression of housekeepers was stable across treatments for both cell lines (P > 0.05). Bars represent the mean ± SEM, and dots represent individual replicates. Data are presented as the quantification cycle (Cq). * P ≤ 0.05 compared to medium alone controls following Tukey’s test.

## Declaration of interest

The authors declare that there is no conflict of interest that could be perceived as prejudicing the impartiality of the research reported.

## Funding

Research reported in this publication was in part supported by the Eunice Kennedy Shriver National Institute of Child Health & Human Development of the National Institutes of Health under Award Number R01HD084316. The content is solely the responsibility of the authors and does not necessarily represent the official views of the National Institutes of Health.
