# Supplementary material for: Bovine endometrial cells do not mount an inflammatory response to Leptospira
Source: Reprod Fertil. 2021 Jul 13;2(3):187–98. doi: 10.1530/RAF-21-0012 (PMC8801031; doi:10.1530/RAF-21-0012)
Supplement: Supplemental Figure 1. Expression of housekeeping genes in BEND and THP-1 cells. Expression of BEND cells GAPDH according to treatment (A-D) and expression of THP-1 cells ACTB according to treatment (E-H). Expression of housekeepers was stable across treatments for both cell lines (P > 0.05). Bars r [file supplementary_figure_1.pdf]

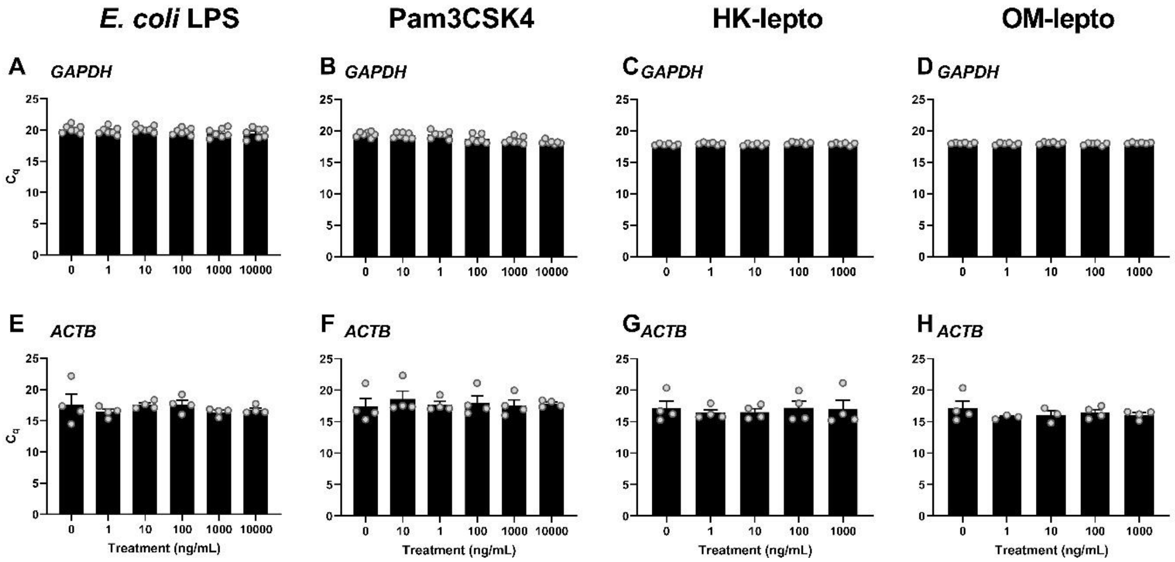

Supplemental Figure 1. Expression of housekeeping genes in BEND and THP-1 cells. Expression of BEND cells *GAPDH* according to treatment (A-D) and expression of THP-1 cells *ACTB* according to treatment (E-H). Expression of housekeepers was stable across treatments for both cell lines ($P > 0.05$). Bars represent the mean ± SEM, and dots represent individual replicates. Data are presented as the quantification cycle (C_q). * $P \leq 0.05$ compared to medium alone controls following Tukey's test.
